# Adult Attachment Patterns Modify the Association Between Social Support and Psychological Distress

**DOI:** 10.3389/fpubh.2018.00249

**Published:** 2018-09-11

**Authors:** Masashi Kizuki, Takeo Fujiwara

**Affiliations:** Department of Global Health Promotion, Tokyo Medical and Dental University, Tokyo, Japan

**Keywords:** Japan, kessler psychological distress scale (K6), internet survey, social support, adult attachment pattern

## Abstract

**Introduction:** Social support is an important protective factor for psychological distress, and adult attachment patterns—which are the basis of human relationships—may modify the association between social support and psychological distress. The objective of this study was to investigate whether adult attachment patterns modify the association between social support and psychological distress.

**Methods:** A commercial online survey service was used to collect data from 1648 men and women of 30–69 years of age in Japan. We assessed the association between social support and psychological distress, as measured by the Kessler Psychological Distress Scale (K6), and stratified it by adult attachment patterns using multiple linear models. Adjustments were made for age, sex, presence of a spouse and child in the household, level of education, employment, and household income.

**Results:** There was a significant interaction effect between social support score and a dismissing attachment pattern on psychological distress (*p* = 0.015); social support was associated with reduced level of psychological distress only in participants with a secure attachment pattern (β:−0.86, 95% CI: −1.56 to −0.16), whereas the point estimate was of opposite sign in participants with a dismissing attachment pattern (β:1.02, 95% CI: −0.32 to 2.37).

**Conclusions:** Higher social support reduced the risk of distress among participants with secure attachment. On the contrary, social support can be harmful for those with a dismissing attachment pattern. Our results suggest that further assessment of adult attachment patterns is needed to maximize the positive effects of social support to prevent psychological distress.

## Introduction

Social support is often conceptualized as “the resources provided by other persons”(1). It is well established in the literature that a high level of social support is protective for depression (2, 3). For example, a higher level of perceived emotional and instrumental support has been associated with a decreased risk for depressive symptoms (4). Researchers have suggested direct influence and/or stress buffers as the pathways by which social support leads to better mental health status (5). Providing social support is considered a low-cost intervention in the community for individuals with low level of social support (6). However, social support does not always show protective effects for psychological distress. Previous studies have discussed the potential variation in the effect of social support on mental health by individual characteristics such as age, gender, and socioeconomic status (4, 7).

A few observational studies have reported an effect modification between social support and adult attachment patterns as predictors of psychological distress (8, 9). Attachment theory suggests that an individual's ability to form close relationships with people is developed mainly during the early years of life, and is categorized into four attachment styles: secure, dismissing, preoccupied, and fearful (10–14). People with secure attachment patterns have a positive view of both oneself and others, whereas those with a fearful attachment pattern have negative views of both oneself and others. Moreira et al. found that “preoccupation reduces the effect of intimate support, while avoidance enhances the effect of casual support” on psychological distress (9). Girme et al. suggested that low-to-moderate practical support from one's partner increased the risk of distress among avoidant participants, whereas all levels of practical support from the partner reduced the risk among non-avoidant participants (8). In addition, an experimental study showed that participants who received verbal support from their partner and had secure attachment patterns presented the lowest anxiety levels in a stress test (15).

Psychological distress is a broad concept that includes mental illness such as psychotic disorder, depressive illness, and anxiety disorder, together with sub-threshold mental disorders that are not identified through specific diagnostic symptoms or signs but by self-rated conditions (16). The impact of social support on psychological distress is known to differ by cultural factors, (17) and past research has also demonstrated that distribution of attachment patterns differs by societies (18). However, no studies to date have examined the interaction effect between social support and adult attachment patterns on psychological distress in Japan. Therefore, in this study, we examined the association between social support and psychological distress by adult attachment pattern among Japan's general population.

## Materials and methods

### Sample

We used the online survey service “Quick Mill” (Macromill, Inc., Tokyo, Japan) to collect data. At the time the survey was conducted, the company maintained 1.2 million users or “monitors” who had registered with a questionnaire survey website (http://monitor.macromill.com/) managed by the company (as of 1st February, 2016). The company continuously recruits monitors from a variety of population groups, mainly through online advertisements on Google, Yahoo! Japan, and other popular websites, e-mail magazines, and printed newspapers and magazines. Registering as a monitor is voluntary and free of charge. Potential monitors must be 6 years or older and not currently working for a market research company or an advertising agency.

Macromill monitors receive e-mails every day informing them of relevant surveys in which they can participate. A monitor can also check a list of available surveys on the personal page of the survey website. When a monitor clicks on a link to an online questionnaire, a description of the survey and an invitation message appear. A monitor can begin the online questionnaire by clicking on the start button. Respondents must answer all questions to finish the survey session, meaning that missing data cannot be generated. After completion of the survey, a monitor will receive points that can be used as electronic currency or exchanged to Japanese yen or gifts. Monitors can respond to the questionnaire either via a personal computer or smartphone.

We set a target sample size of 1600 participants, comprised of 200 respondents from each of the 8 age- and sex-specific population strata (males and females aged 30–39, 40–49, 50–59, and 60–69 years). A random sample of eligible monitors was drawn from each age- and sex-specific population stratum, and selected monitors were invited to participate in the survey. The number of monitors invited to take part in the survey was determined using computer algorithms and continuously increased based on the remaining number of responses needed to be collected. The recruitment was ended when 212 monitors, including reserve data for 12 additional monitors (6% of the intended number, according to the company's specification), completed the questionnaire in each stratum.

### Psychological distress measurement

The main outcome was psychological distress measured by the Kessler Psychological Distress Scale (K6) (19), which consists of six items, with each being scored from 0 to 4. The total score ranges from 0 to 24, with higher scores indicating more severe distress. The scale has excellent internal consistency reliability among Japanese adults [α = 0.88 among factory workers (20) and α = 0.85 among samples including community residents and psychiatric outpatients (21)]. Additionally, the K6 score is significantly higher among psychiatric outpatients with mood/anxiety disorder compared with community residents (21), suggesting good validity.

### Social support measurement

The main predictor was perceived social support measured by the Jichi Medical School Social Support Scale (JMS-SSS) (22). The original version of the JMS-SSS separately assesses the availability of functional support from the spouse, family, and friends using the same set of items. The JMS-SSS has excellent internal consistency reliability (spouse: α = 0.89, family: α = 0.95, and friends: α = 0.94) (22). The all JMS-SSS scores are significantly associated with an increased odds of sleep disturbance in older persons (23), showing good validity. In this study, we combined spouse and other family members into the “family” category and assessed support from them together. Respondents answered 10 questions about their relationships with family and friends (1, strongly disagree, 2, somewhat disagree, 3, somewhat agree, and 4, strongly agree). Total scores were calculated by combining scores for family and friends, and categorized into two groups: low social support (≤ median) and high social support (> median).

### Attachment pattern measurement

General attachment patterns were assessed using the Relationship Structures (ECR-RS) questionnaire (24), further modified by Fraley (25) to measure feelings about close relationships in general. ECR-RS produces two scores: the attachment-related avoidance score and the attachment-related anxiety score (26). The original ECR-RS has excellent internal consistency reliability for mother, father, partner, and best friend (anxiety scale: α = 0.88, 0.90, 0.91, and 0.90, respectively, and avoidance scale: α = 0.92, 0.90, 0.87, and 0.88, respectively) and the pattern of correlations with the Big Five personality traits are the same for ECR-RS and the longer Experiences in Close Relationships-Revised (ECR-R) questionnaire (24). The Japanese language version of ECR-RS has good internal consistency reliability (anxiety scale: ω = 0.75–0.83, and avoidance scale: ω = 0.88–0.95) and the scores were significantly associated with Internal Working Model Scale, Relationship Questionnaire, and the Experiences in Close Relationships inventory-the-generalized-other-version (27). The reliability and validity of ECR-RS about close relationships in general has not been reported; however, the global scores created based on context-specific scores showed excellent internal consistency reliability (anxiety: α = 0.85, and avoidance: α = 0.88) and were significantly associated with ECR-R (24). After attachment-related avoidance score and the attachment-related anxiety score in reference to others were calculated, the attachment patterns of each sample were categorized into four groups as follows: as “secure” if both anxiety and avoidance scores were the same or lower than the median; “dismissing” if the anxiety score was the same or lower than the median and the avoidance score was higher than the median; “preoccupied” if the anxiety level was higher than the median and avoidance score was the same or lower than the median, and “fearful” if both the anxiety and avoidance scores were higher than the median.

### Covariates

We collected covariates including age (in years), sex, presence of a spouse, and a child in the household, level of education (categorized as < university and ≥ university), employment, and household income.

### Analysis

We used linear regression to examine the association between categorical social support scores and psychological distress score, and interactions term between social support and attachment patterns. All covariates were included in the model to control for individual-level confounders. Significance of each interaction term was assessed using the Wald test. To facilitate interpretation, we also applied stratified analysis for each attachment pattern, and reported the attachment-pattern-specific partial regression coefficients for social support.

### Ethical considerations

This study was approved by the Institutional Review Board at Tokyo Medical and Dental University (M2015-509).

## Results

A total of 9938 monitors were invited to take the survey. The number of invited monitors in each stratum ranged from 658 (females aged 50–59 years) to 1713 (males aged 30–39 years). The survey was completed within a day and we obtained data for 1648 respondents (206 samples in each stratum) from Macromill, Inc. and used all the samples in the analyses.

Table 1 presents the frequency distribution of participants' socioeconomic characteristics. The proportion of respondents who had graduated from university was significantly higher in this study than the 2015 Population Census for all 8 age- and sex-specific population strata (all *p* < 0.001 by one-sample test for a binomial proportion). The distribution of household income in this study was not significantly different from the 2014 National Survey of Family Income and Expenditure (*p* = 0.07 by the Cochran-Armitage test for trend). Using our operational definitions of attachment patterns, 646 (39.2%) participants were categorized as secure, 341 (20.7%) as dismissing, 263 (16.0%) as preoccupied, and 398 (24.2%) as fearful. Significant differences were observed in social support by attachment patterns (*p* < 0.001 by chi-square test). The percentage of higher than median social support scores was 64.9% for secure, 28.7% for dismissing, 64.3% for preoccupied, and 28.4% for fearful attachment patterns.

**Table 1:** Socioeconomic characteristics of participants.

	***N***	(*%*)
Living with a spouse		
Yes	1097	(66.6)
No	551	(33.4)
Living with a child		
Yes	721	(43.8)
No	927	(56.3)
Graduated from university		
Yes	919	(55.8)
No	729	(44.2)
Employment status		
Employed^*^	1182	(71.7)
Unemployed	466	(28.3)
Annual household income		
<2 million yen	131	(7.9)
2–<4 million yen	350	(21.2)
4–<6 million yen	335	(20.3)
6–<8 million yen	213	(12.9)
8–<10 million yen	133	(8.1)
≥10 million yen	136	(8.3)
Missing^†^	350	(21.2)

* *Employed category includes temporal leave for child and/or elderly care and/or childbirth*.

† Either “I don't know” or “I don't want to answer.”

The level of psychological distress stratified by attachment patterns and level of social support is shown in Table 2. Overall, the mean [standard deviation (SD)] K6 score was 6.1 (5.9). The mean K6 scores were significantly higher in participants with higher level of social support (*p* < 0.001 by *t*-test), and significantly different between participants with different attachment patterns (*p* < 0.001 by analysis of variance, and *p* < 0.001 for all multiple-comparison tests after Bonferroni correction).

**Table 2:** Kessler psychological distress scale score by attachment pattern and level of social support.

	***N***	**Mean (SD)**
Attachment pattern^*^		
Secure	646	3.9 (4.4)
Dismissing	341	5.6 (5.7)
Preoccupied	263	7.9 (6.5)
Fearful	398	8.8 (6.3)
Social support^†^		
> Median score	849	5.6 (5.8)
≤ Median score	799	6.6 (5.9)

* *Attachment-related anxiety and avoidance were measured by the Experiences in Close Relationships-Relationship Structures (ECR-RS) scale. Participants were categorized as follows: “secure” if both scores were the same or lower than the medians; “dismissing” if the anxiety score was the same or lower than the median and the avoidance score was higher than the median; “preoccupied” if the anxiety score was higher than the median and the avoidance score was the same or lower than the median, and “fearful” if both scores were higher than the median*.

† *Social support was measured by the Jichi Medical School Social Support Scale and categorized into higher than the median and the same or lower than the median*.

Table 3 shows the results of multiple regression analyses. Social support was associated with decreased level of psychological distress (β −0.79, 95% CI: 1.34 to −0.23) when attachment patterns were not taken into account (Model 1). There was a significant interaction effect between social support and a dismissing attachment pattern (*p* = 0.015).

**Table 3:** Multiple regression models of the influence of social support^†^ on Kessler Psychological Distress Scale (K6) score with and without adult attachment pattern^‡^.

	**Model 1**		**Model 2**	
	**β**	**95% CI**	**β**	**95% CI**
> Median social support score	−0.79^**^	(−1.34, −0.23)	−0.84	(−1.72, 0.03)
Attachment pattern (ref: secure)				
Dismissing			0.62	(−0.36, 1.60)
Preoccupied			3.06^***^	(1.76, 4.36)
Fearful			3.77^***^	(2.81, 4.73)
Social support × Attachment pattern (ref: secure)				
> Median social support score × Dismissing			1.91^*^	(0.38, 3.45)
> Median social support score × Preoccupied			0.65	(−0.96, 2.26)
> Median social support score × Fearful			0.66	(−0.80, 2.12)
Age	−0.13^***^	(−0.15, −0.10)	−0.10^***^	(−0.13, −0.08)
Female	0.09	(−0.48, 0.66)	0.67^*^	(0.12, 1.23)
Living with a spouse	−0.62	(−1.32, 0.08)	−0.47	(−1.14, 0.21)
Living with a child	−0.55	(−1.18, 0.09)	−0.56	(−1.16, 0.04)
Graduated from university	−0.54	(−1.10, 0.03)	−0.19	(−0.73, 0.35)
Employed	−0.26	(−0.91, 0.39)	−0.04	(−0.67, 0.58)
Annual household income (ref: <2 million yen)				
2–<4 million yen	−1.27^*^	(−2.42, −0.11)	−0.88	(−1.99, 0.22)
4–<6 million yen	−2.46^***^	(−3.66, −1.25)	−1.79^**^	(−2.95, −0.63)
6–<8 million yen	−1.83^**^	(−3.13, −0.53)	−1.25^*^	(−2.50, −0.01)
8–<10 million yen	−2.34^**^	(−3.77, −0.92)	−1.67^*^	(−3.04, −0.29)
≥10 million yen	−2.13^**^	(−3.58, −0.68)	−1.77^*^	(−3.15, −0.38)

*** p <0.001;

** p <0.01;

* *p <0.05*.

*Results for missing category of annual household income are not shown*.

† *Social support was measured by the Jichi Medical School Social Support Scale and categorized into higher than the median and the same or lower than the median*.

‡ *Attachment-related anxiety and avoidance were measured by the Experiences in Close Relationships-Relationship Structures (ECR-RS) scale. Participants were categorized as follows: “secure” if both scores were the same or lower than the medians; “dismissing” if the anxiety score was the same or lower than the median and the avoidance score was higher than the median; “preoccupied” if the anxiety score was higher than the median and the avoidance score was the same or lower than the median, and “fearful” if both scores were higher than the median*.

Table 4 shows the results of multiple regression analyses, stratifying by attachment pattern. Social support showed a protective effect on the risk of psychological distress in participants with secure attachment patterns (β −0.86, 95% CI: −1.56 to −0.16). In contrast, social support increased the level of psychological distress in participants with a dismissing attachment pattern (β 1.02, 95% CI: −0.32 to 2.37), although this relationship was not significant (*p* = 0.14). Social support was not associated with psychological distress in participants with preoccupied and fearful attachment patterns (*p* = 0.74 and *p* = 0.99, respectively).

**Table 4:** Multiple regression models of the influence of social support^†^ on Kessler Psychological Distress Scale (K6) score by attachment pattern^‡^.

	**Attachment pattern**							
	**Secure**		**Dismissing**		**Preoccupied**		**Fearful**	
	**β**	**95% CI**	**β**	**95% CI**	**β**	**95% CI**	**β**	**95% CI**
> Median social support score	−0.86^*^	(−1.56, −0.16)	1.02	(−0.32, 2.37)	−0.27	(−1.88, 1.34)	0.01	(−1.35, 1.37)
Age	0.57	(−0.16, 1.29)	0.14	(−1.13, 1.41)	0.68	(−0.96, 2.32)	1.44^*^	(0.16, 2.71)
Female	−0.06^***^	(−0.09, −0.03)	−0.12^***^	(−0.18, −0.06)	−0.14^***^	(−0.21, −0.07)	−0.13^***^	(−0.19, −0.07)
Living with a spouse	0.68	(−0.23, 1.59)	−0.85	(−2.27, 0.58)	−1.13	(−3.11, 0.85)	−0.81	(−2.38, 0.76)
Living with a child	−0.19	(−0.94, 0.55)	0.76	(−0.65, 2.17)	−2.04^*^	(−3.84, −0.25)	−1.00	(−2.52, 0.52)
Graduated from university	0.31	(−0.39, 1.00)	0.14	(−1.11, 1.39)	−0.27	(−1.93, 1.38)	−1.08	(−2.31, 0.16)
Employed	0.41	(−0.41, 1.22)	−1.28	(−2.76, 0.20)	0.65	(−1.22, 2.51)	0.15	(−1.26, 1.56)
Annual household income (ref: <2 million yen)								
2–<4 million yen	−1.12	(−3.12, 0.88)	−1.46	(−3.68, 0.76)	1.83	(−1.27, 4.92)	−1.58	(−3.80, 0.64)
4–<6 million yen	−2.75^**^	(−4.80, −0.71)	−1.94	(−4.25, 0.37)	0.60	(−2.56, 3.75)	−1.97	(−4.44, 0.50)
6–<8 million yen	−1.58	(−3.70, 0.54)	−1.87	(−4.56, 0.82)	−0.41	(−3.77, 2.95)	−1.15	(−3.80, 1.51)
8–<10 million yen	−2.42^*^	(−4.61, −0.22)	−0.99	(−3.81, 1.82)	0.92	(−3.23, 5.08)	−3.12	(−6.27, 0.02)
≥10 million yen	−2.58^*^	(−4.83, −0.33)	−2.13	(−5.09, 0.83)	2.00	(−1.62, 5.62)	−3.82^*^	(−7.10, −0.55)

*** p <0.001;

** p <0.01;

* *p <0.05*.

*Results for missing category of annual household income are not shown*.

† *Social support was measured by the Jichi Medical School Social Support Scale and categorized into higher than the median and the same or lower than the median*.

‡ *Attachment-related anxiety and avoidance were measured by the Experiences in Close Relationships-Relationship Structures (ECR-RS) scale. Participants were categorized as follows: “secure” if both scores were the same or lower than the medians; “dismissing” if the anxiety score was the same or lower than the median and the avoidance score was higher than the median; “preoccupied” if the anxiety score was higher than the median and the avoidance score was the same or lower than the median, and “fearful” if both scores were higher than the median*.

## Discussion

Our main finding suggests that the magnitude of the effects of perceived support on psychological distress varied across adult attachment patterns. In particular, social support was protective only in participants with secure attachment patterns. The beneficial effect of social support on mental well-being was not observed in participants with insecure attachment patterns (dismissing, preoccupied, and fearful). Moreira et al. (9) speculated that preoccupied individuals regarded support provision to be “less sincere, less selfless and less likely to be consistently provided in the future,” which might reduce the effect of support within close relationships among preoccupied individuals (9). Girme et al. described the negative responses in avoidant individuals when receiving low-to-moderate levels of practical support as “automatic self-protective strategies that help bypass the vulnerability of depending on (what avoidant people expect will be) unreliable caregivers” (8). A study by Collins and Feeney found that individuals with high levels of avoidance were more likely to negatively evaluate their partner's attempts at giving support (28). These findings reinforce the idea that attachment patterns influence how individuals perceive social support.

It is also widely accepted that interpersonal relationships are modified by individual characteristics (29). Kuscu et al. reported that unstable attachment patterns were associated with lower perceived social support from family and friends (30). Levels of social support were lower in participants with dismissing and fearful attachment patterns in this study. However, evidence showed that social support is not the primary mediator of the association between attachment patterns and mental health, even though the mechanism is theoretically indefensible (9). In this study, the inclusion of two-way interaction terms between attachment patterns and social support also did not substantially alter the magnitude of the association between attachment patterns and psychological distress in the multivariable models (*p* = 0.20 using likelihood-ratio test, results not shown).

Adult attachment patterns are related to the infant-caregiver relationship (31). Hazan and Shaver found that adults who were secure in their romantic relationships, in comparison with insecure adults, reported warmer relationships with both parents (32). In a summary of published studies on attachment patterns, Fraley (33) found a moderate degree of stability (equivalent to *r* = 0.25–0.39) in attachment from infancy to adulthood (33). In this study, the mediation effect of the attachment pattern on the association between social support and mental health, although not statistically significant, is an example of how circumstances early in the life course can affect adult health in later life.

The direction of the social support coefficient suggests that higher level of social support can have a deleterious mental health effect in participants with a dismissing attachment pattern (highly avoidant but not anxious), although the association was not statistically significant. There may be several explanations for the possible positive association between social support and high risk of psychological distress among adults with a dismissing attachment pattern. As hypothesized by Girme et al. avoidant persons prefer to be rigidly self-reliant in order to prevent problems that may arise from relying on unreliable support, and low-to-moderate support may even create fear of dependence and increase distress (8). Rholes et al. found higher levels of depression among avoidant parents after birth when they perceived more partner support, more closeness, or less partner negativity, and suggested that “highly avoidant people may experience more depressive symptoms when their independence is threatened or compromised” and “fear being subjected to the pressure of becoming long-term caretakers” (34). According to these discussions, participants with dismissing attachment patterns in our sample might recognize higher levels of social support as a threat to self-reliance and a burden, rather than emotional relief and/or a chance to increase their overall life satisfaction.

Another potential explanation includes reverse causation. Since this study used cross-sectional data, it is possible that higher levels of social support are the outcome of the association, where avoidant and depressed participants require support from family and friends in order to live in the community. This will be a subject for further longitudinal investigation.

This study has several limitations. First, participants were monitors of an online survey service and represented only those populations who used the website during the study period. Macromill reported that the proportion of married participants and their household income was similar to the general population; however, the monitors were slightly more likely to live in a small household, to live in an apartment, and to be a housewife, and less likely to have social interactions with neighbors, and to be satisfied with current daily lives compared with the general population (35–38). Therefore, it is likely that participants had a lower level of social support and a higher risk of psychological distress due to anxiety in their daily lives compared to the general population. However, these differences do not necessarily lead to bias in the effect measures (39). Second, social support is often divided into emotional, instrumental, appraisal, and informational support, (40–42) but the JMS-SSS was initially designed to capture only emotional, instrumental, and informational support (22). However, behaviors related to emotional, appraisal, and informational support often overlap (40) and failure to assess appraisal support is less likely to influence the results. Third, ECR-RS about close relationships in general has not been assessed in Japanese population, and the reliability of validity of the instrument used in the present study has not been confirmed. It is possible that the variation in the relationship between social support and psychological distress according to attachment pattern had been underestimated due to potential misclassification of adult attachment patterns. Fourth, as this study uses cross-sectional data, we cannot determine the direction of the association between social support and psychological distress. The general agreement among researchers that social support influences psychological distress was applied to our interpretation of the results. In contrast, the stability of the attachment pattern was moderate, the attachment pattern was modifiable during the life course, (31) and social support and psychological distress in adulthood may influence the adult attachment pattern. Longitudinal examinations are particularly important for causal inference in the modifying effect of adult attachment patterns on social support on mental health. Fifth, some studies showed different effects of social support on mental health between men and women (43). As the interaction between sex and social support was not significant in our sample (*p* = 0.87 using likelihood-ratio test, results not shown), we did not stratify the results by sex.

In summary, a higher level of perceived support from family and friends was associated with a lower risk of psychological distress among adults on average; however, the directions and strengths of the association might differ according to adult attachment patterns. Perceived support reduced the risk of distress among participants with secure attachment. On the contrary, higher perceived support can be harmful for those with dismissing attachment patterns. Our results suggest that future studies of social support should include the assessment of adult attachment patterns.

## Author contributions

MK designed the survey, analyzed data, and drafted the manuscript; and TF reviewed results and revised the manuscript.

### Conflict of interest statement

The authors declare that the research was conducted in the absence of any commercial or financial relationships that could be construed as a potential conflict of interest.
